# Meeting of the Henry County Medical Society

**Published:** 1858-03

**Authors:** N. Holton, T. D. Fitch

**Affiliations:** President; Secretary


### PROCEEDINGS OF MEDICAL SOCIETIES.

---

### MEETING OF THE HENRY COUNTY MEDICAL SOCIETY, HELD IN CAMBRIDGE, HENRY CO., DEC. 1, 1857.

President Holton in the chair.

Minutes of the previous meeting, held in Geneseo, Sept. 1st, 1857, were read and approved.

On motion of Dr. R. J. Stough, a vote of thanks was paid Dr. A. A. Dunn for his address made at Geneseo at our last meeting.

Committee appointed to obtain meteorological instruments reported progress.

On motion of Dr. M. J. Lyman, report was accepted, and committee continued.

On motion of Dr. R. J. Stough, an equal assessment will be made on the members of the society, to pay for the aforesaid instruments.

Dr. Swansey, of Tiskilwa, was proposed by Dr. N. Holton, and on motion, Dr. Swansey will be admitted by complying with the regulations of our constitution and by-laws.

Essays and reports of cases.

Dr. L. P. Dimmitt reported several cases of cutaneous diseases, treated by the following formulæ:

| | | | |
|---|---|---|---|
| ℞ | Lard oil, | ℥vi. | |
| | Oil turpentine, | ℥ij. | |
| | Acid sul., | ℥i. | M. |

Dr. R. J. Stough read a report of a case of dysentery, treated principally by the cannabis indica (successfully) by Dr. Talcott.

Dr. M. J. Lyman on chronic pleuritis, treated by veratrum viride, following which was a lengthy discussion upon the use and abuse of veratrum.

Dr. R. J. Stough read an essay upon the use of belladonna as a prophylactic in scarlatina.

Dr. E. Pomeroy read an essay, entitled "Some thoughts relative to the medical profession," confined chiefly to medical progress.

Dr. T. D. Fitch reported a case of laceration of the neck and inversion of the uterus—reduction and recovery.

Other cases were reported verbally, and discussed.

On motion, the address of Dr. N. Holton, to be delivered at the evening session, was deferred, on account of the inclemency of the weather, until our next regular meeting.

On motion of Dr. R. J. Stough, society adjourned, to meet in Sheffield, Bureau Co., on first Tuesday in March, 1858.

N. HOLTON, *President.*

T. D. Fitch, *Secretary.*

